# Incremental value of serum neurofilament light chain and glial fibrillary acidic protein as blood-based biomarkers for predicting functional outcome in severe acute ischemic stroke

**DOI:** 10.1177/23969873241234436

**Published:** 2024-02-24

**Authors:** Christoph Vollmuth, Cornelia Fiessler, Felipe A Montellano, Alexander M Kollikowski, Fabian Essig, Patrick Oeckl, Lorenzo Barba, Petra Steinacker, Cara Schulz, Kathrin Ungethüm, Judith Wolf, Mirko Pham, Michael K Schuhmann, Peter U Heuschmann, Karl Georg Haeusler, Guido Stoll, Markus Otto, Hermann Neugebauer

**Affiliations:** 1 University Hospital Würzburg, Department of Neurology, Würzburg, Germany; 2 University of Würzburg, Institute for Clinical Epidemiology and Biometry, Würzburg, Germany; 3 University Hospital Würzburg, Department of Neuroradiology, Würzburg, Germany; 4 University Hospital Ulm, Department of Neurology, Ulm, Germany; 5 German Center for Neurodegenerative Diseases e.V. (DZNE) Ulm, Ulm, Germany; 6 Martin-Luther-University of Halle-Wittenberg, Department of Neurology, Halle (Saale), Germany; 7 Institute for Medical Data Science, University Hospital Würzburg, Würzburg, Germany; 8 Clinical Trial Centre, University Hospital Würzburg, Würzburg, Germany

**Keywords:** Blood-based biomarkers, NfL, GFAP, prediction, prognosis, ischemic stroke

## Abstract

**Introduction:**

Blood-based biomarkers may improve prediction of functional outcome in patients with acute ischemic stroke. The role of neurofilament light chain (NfL) and glial fibrillary acidic (GFAP) as potential biomarkers especially in severe stroke patients is unknown.

**Patients and Methods:**

Prospective, monocenter, cohort study including consecutive patients with severe ischemic stroke in the anterior circulation on admission (NIHSS score ⩾ 6 points or indication for mechanical thrombectomy). Outcome was assessed 3 months after the index stroke by the modified Rankin Scale (mRS). Serum biomarkers levels of NfL and GFAP were determined by ultrasensitive ELISA. Univariate and multivariate logistic regression models were performed to determine the association of biomarker levels and functional disability. Discrimination, calibration, and overall performance were analyzed in different models via AUROC, calibration plots (with Emax and Eavg), Brier-score and R2 using variables, identified as important covariates for functional outcome in previous studies.

**Results:**

Between 06/2020 and 08/2021, 213 patients were included [47% female, mean age 76 (SD ± 12) years, median NIHSS score 13 (interquartile range, IQR 9; 17)]. Biomarker serum levels were measured at a median of 1 [IQR, 1; 2] day after admission. Compared to patients with mRS 0–2 at 3 months, patients with mRS 3–6 had higher serum levels of NfL (median: 136 pg/ml vs 41 pg/ml; *p* < 0.0001) and GFAP (700 ng/ml vs 9.6 ng/ml; *p* < 0.0001). Both biomarkers were significantly associated with functional outcome [adjusted logistic regression, odds ratio (95% CI) for NfL: 2.63 (1.62; 4.56), GFAP: 2.16 (1.58; 3.09)]. In all models the addition of serum NfL led to a significant improvement in the AUROC, as did the addition of serum GFAP. Calibration plots showed high agreement between the predicted and observed outcomes and after addition of the two blood-based biomarkers there was an improvement of the overall performance.

**Conclusion:**

Prediction of functional outcome after severe acute ischemic stroke was improved by the blood-based biomarkers serum NfL and GFAP, measured in the acute phase of stroke. These findings have to be replicated in independent external cohorts.

Study registration: DRKS00022064

## Graphical abstract

**Graphical abstract:**
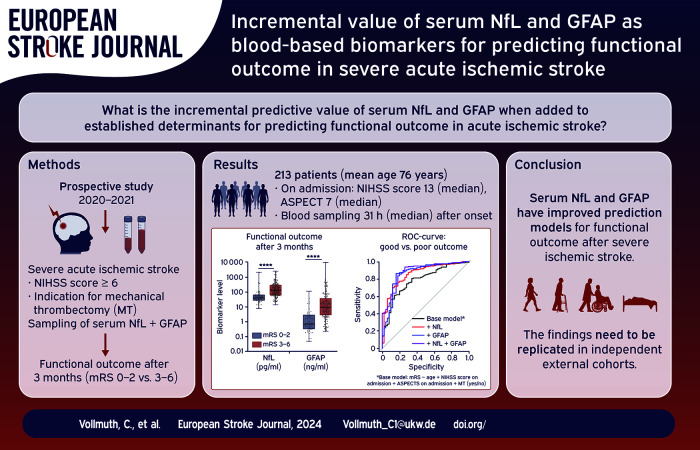

## Introduction

Outcome after severe ischemic stroke widely varies from complete recovery to severe functional impairment or death.^1,2^ While factors such as stroke severity, infarct volume and comorbidities are associated with functional outcome, their predictive values for estimation of long-term functional outcome are limited.^3^ Hence, blood-based biomarkers, which can easily be obtained, could provide additional information on the extent of ischemic brain damage and may support estimation of functional outcome.^4^

One potential prognostic blood-based biomarker is neurofilament light chain protein (NfL). NfL is a valuable surrogate marker for neuroaxonal injury and has been shown to be associated with short- and long-term outcomes in stroke patients.^5–8^ Another potential blood-based biomarker, glial fibrillary acidic protein (GFAP) has been explored as a surrogate of astrocytic cell death.^9–11^ Recent studies have demonstrated an association between serum NfL and GFAP levels and functional outcome in ischemic stroke patients.^5–8^ Nevertheless, the extent to which these two biomarkers enhance the prediction of functional outcomes in patients with severe acute ischemic stroke beyond established determinants, remains uncertain. Moreover, there are presently no established predictive models integrated into clinical practice.

The aim of this study was to investigate the association between serum NfL and GFAP on hospital admission and the functional outcome 3 months after severe acute ischemic stroke. Furthermore, we aimed to determine the incremental value of these blood-based biomarkers on top of a prognostic model in these stroke patients.

## Methods

In this prospective, monocenter observational cohort study, we enrolled patients presenting with severe acute ischemic stroke in the anterior circulation, characterized by a National Institutes of Health Stroke Scale (NIHSS) score of ⩾6 points upon admission and/or an indication for mechanical thrombectomy, that is, proximal cerebral artery vessel occlusion irrespective of NIHSS. Exclusion criteria were age under 18 years and insufficient German language. Furthermore, we excluded those who had previously taken part in trials involving tracking devices or surgical procedures that could potentially affect platelet function or stroke outcomes within the past 3 months.

### Ethical approval

The study was conducted in accordance with the Declaration of Helsinki and its recent modifications. Written informed consent was obtained from all participants or legal representatives. This study was approved by the local Ethics Committee of the University of Würzburg (reference n. 05/20-am) and was registered (DRKS00022064).

### Clinical variables

Data were collected in a central database. All demographic and clinical variables were registered systematically. In detail, data on the NIHSS score on admission, after 24, 48, and 72 h as well as at hospital discharge were assessed by experienced neurologists. Furthermore, the Alberta Stroke Program CT Score (ASPECTS) on admission^12^ and 24–72 h after stroke onset, the collateral status^13^ and the expanded treatment in cerebral ischemia (eTICI) score were assessed by independent experienced neuroradiologists.^14^ Etiology of ischemic stroke was based according to the trial of ORG 10172 in acute stroke treatment (TOAST) classification on information available at discharge.^15^ Neurologic disability was assessed before admission and at hospital discharge using the modified Rankin Scale (mRS). Functional outcome 3 months (±14 days) after index stroke was assessed by structured telephone interview by a blinded rater. A good functional outcome was defined as a mRS 0–2 and a poor outcome was defined as mRS 3–6. Furthermore, cardiovascular risk factors and diseases were determined by patients interview and chart review. In addition, short term outcome was investigated as a secondary outcome and defined as NIHSS in the time course, NIHSS progression within 1 day and mortality (Supplement).

### Blood sampling and analysis

Blood was drawn on the morning after enrollment from the antecubital or femoral vein, spun down at 2500 × *g* for 10 min for serum generation, snap frozen and stored at −80°C until analysis. All biomarker analyses were performed according to the manufacturers’ instructions by experienced operateurs who were blinded to clinical information of the patients. In detail, GFAP serum levels were determined using a commercially available digital immunoassay by using an HD-X Simoa machine (Quanterix Inc., Lexington, USA). NfL serum levels were measured with a commercial kit for the automated ELLA microfluidic system (BioTechne, Minneapolis, USA).

### Statistical analysis

Statistical analysis was performed using R 4.2.2 (R Foundation, Vienna, Austria) and GraphPad 8 (GraphPad Software, La Jolla, USA). We tested differences between groups using the χ^2^ test, Student’s *t*-test, and Mann–Whitney *U* test, according to the distribution of the variables. Categorical variables were reported as numbers of patients with percentage of the total cohort (%) and continuous variables with normal distributions were reported as mean with standard deviation (SD), while non-normally distributed variables were presented as median with interquartile range (IQR). Normality of distributions was assessed graphically. Biomarker levels were logarithmically transformed. Correlations between biomarker levels were calculated with the Spearman’s coefficient. Associations of blood-based biomarkers with poor outcome was assessed using logistic regression. Adjustment was conducted for variables that have been identified as the most important covariates [age, NIHSS on admission, ASPECTS on hospital admission (0–7vs 8–10), systemic thrombolysis (yes/no), mechanical thrombectomy (yes/no) and pre-stroke mRS (0–2 vs 3–5)] for poor outcome after ischemic stroke in previous studies according to current statistical guidance.^16^ Odds ratios (ORs) were reported with 95% confidence intervals (95% CI). Statistical significance was determined if the *p* value was less than 0.05. All tests were performed two-tailed.

Three baseline prognostic models were generated including age and NIHSS on admission as previously suggested.^4^ Further, therapeutic interventions and infarct volume in terms of ASPECT Score on admission and ASPECT Score 24–72 h after index stroke were also included, which are known to be associated with functional outcome after ischemic stroke^17^:

**Model A:** mRS ~ age + NIHSS Score on admission.
**Model B:** mRS ~ age + NIHSS Score on admission + ASPECTS on admission + mechanical thrombectomy (yes/no).
**Model C:** mRS ~ age + NIHSS Score on admission + ASPECTS 24–72 h after stroke onset + mechanical thrombectomy (yes/no).

Area under the Receiver Operating Characteristic Curve (AUROC) analysis were constructed for the three models to assess the discriminative value in terms of sensitivity and specificity for good functional outcome. To evaluate the incremental value of the studied biomarkers on top of the three prognostic models (A, B, C), the improvement of the discrimination using the DeLong test was analyzed.^18^ Calibration plots probability were used to depict the observed versus the predicted probability of poor outcome and to report Emax and Eavg as measures of calibration and further the improvement of the calibration was analyzed. The overall model’s performance was assessed using the Brier-score (the higher the better) and the R2 to investigate the percentage of the variance explained by a single or combination of variables.^19^

## Results

### Baseline characteristics and outcome after 3 months

**Figure 1.**
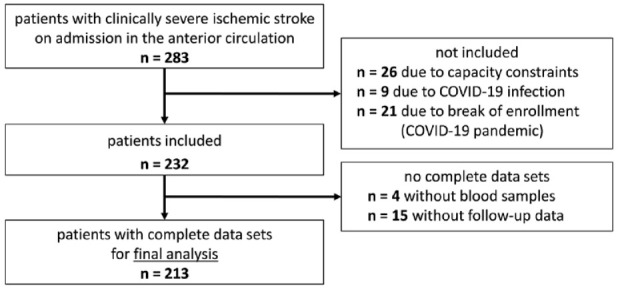
Final study population selection process.

Between 06/2020 und 08/2021, 283 patients with acute ischemic stroke in the anterior circulation were admitted at our tertiary care center (University Hospital Würzburg, Würzburg, Germany) and eligible according to the chosen exclusion and inclusion criteria. In consequence of a break of enrollment due to COVID-19 between December 2020 and January 2021, 21 stroke patients, treated during that period, could not be included in the study. Additional 26 patients could not be included in the study due to capacity constraints and 9 patients due to COVID-19 infection. Of 232 patients included, four patients did not receive blood samples and 15 patients did not participate in follow-up. Thus, a total of 213 patients were analyzed (Figure 1).

Overall, 213 patients [47% female, mean age 76 (SD ± 12) years, median NIHSS score on admission 13 points (IQR, 9; 17), median ASPECTS score on admission 7 (IQR, 6; 9)] with complete biomarker and follow up information were included in the study. A total of 158 (74%) patients underwent mechanical thrombectomy and 85 (40%) systemic thrombolytic therapy, including 57 (27%) patients undergoing both treatments (Table 1).

**Table 1.** Clinical, radiological and biochemical data of the study population.

	Total (*n* = 213)	mRS 0–2 (*n* = 50)	mRS 3–6 (*n* = 163)	*p*-Value*
Baseline data				
Mean age, years (±SD)	76.1 (±12.5)	73.5 (±10.8)	76.9 (±12.9)	0.092
Sex, female, *n* (%)	100 (47.0)	31 (62.0)	69 (42.0)	**0.016**
Pre stroke mRS 0–2, *n* (%)	177 (83.0)	50 (100.0)	127 (78.0)	**0.0005**
Wake-up stroke, *n* (%)	68 (32.0)	12 (24.0)	56 (34.0)	0.22
Mean creatinin, mg/dl (±SD)	1.1 (±0.7)	1.0 (±0.4)	1.1 (±0.7)	0.59
Mean GFR, ml/min/173 cm^2^ (±SD)	71.4 (±27.3)	75.8 (±22.0)	70.0 (±28.6)	0.10
Time from onset to mechanical recanalisation, hours,^a,b^ hours, mean (±SD)	4.3 (±3.1)	3.6 (±1.8)	4.5 (±3.4)	0.40
Vascular risk factors, *n* (%)				
Hypertension	145 (68.1)	32 (64.0)	115 (70.6)	0.30
Diabetes mellitus	42 (19.7)	7 (14.0)	35 (21.5)	0.31
Heart failure	31 (14.6)	4 (8.0)	27 (16.6)	0.17
Atrial fibrillation	99 (46.5)	26 (52.0)	73 (44.8)	0.42
Stroke severity,^c^ median [IQR]				
NIHSS on admission	13 [9; 17]	9.5 [8; 14]	14 [10; 17]	**0.0003**
NIHSS at 24 h	12 [5; 20]	3 [1; 5]	15 [8.5; 22]	<**0.0001**
NIHSS at 72 h	9.5 [3; 16]	2 [0; 4]	13 [7; 19]	<**0.0001**
NIHSS at discharge	5 [1; 12]	1 [0; 3]	9.5 [4; 14]	<**0.0001**
Etiology, *n* (%)				
Large artery atherosclerosis	26 (12.2)	8 (16.0)	18 (11.0)	0.46
Cardioembolism	98 (46.0)	27 (54.0)	71 (43.6)	0.26
Unknown/others	89 (41.8)	15 (30.0)	74 (45.4)	0.07
Neuoradiologic data				
Blood vessel occluded, *n* (%)				
ICA	48 (22.5)	6 (12.0)	42 (25.8)	**0.0094**
M1	99 (46.5)	22 (44.0)	77 (47.2)	**0.0211**
M2	48 (22.5)	13 (26.0)	35 (21.5)	**0.0088**
ASPECTS, median [IQR]				
ASPECTS on admission	7.0 [6.0; 9.0]	8.5 [8.0; 9.0]	7 [6; 8]	<**0.0001**
ASPECTS 24–72 h	7.0 [5.0; 8.0]	8.0 [7.0; 9.0]	6 [3; 7]	<**0.0001**
Collateral status, *n* (%)				
1	95 (44.6)	13 (26.0)	82 (50.3)	**0.0032**
2	56 (26.3)	17 (34.0)	39 (23.9)	0.20
3	32 (15.0)	9 (18.0)	23 (14.1)	0.65
Not assessable	30 (14.0)	11 (22.0)	19 (11.7)	
Acute treatment, *n* (%)				
Systemic lysis	85 (40.0)	24 (48.0)	61 (37.0)	0.20
Mechanical recanalisation	158 (74.0)	32 (64.0)	126 (77.3)	0.07
TICI ⩾ 2b	132 (84.0)	32 (100.0)	100 (79.4)	**0.013**
Biomarker data, median [IQR]				
Time from onset to blood sample, hours^a^	31 [21; 59]	27 [18; 48]	35 [22; 63]	0.07
NfL, pg/ml	96 [51; 228]	41 [29; 62]	136 [67; 277]	<**0.0001**
GFAP, ng/ml	5.7 [1.5; 22]	0.7 [0.3; 2.7]	9.6 [3.0; 33]	<**0.0001**

a Only patients with clear time windows.

b Only patients with mechanical recanalization.

c Deceased patients excluded.

* Patients with good functional outcome versus poor outcome.Bold values refer to statistically significant.

While 50 of 213 (23%) had a good functional outcome (mRS 0–2), 163 of 213 (77%) patients had a poor functional outcome (mRS 3–6), including 81 patients (38.0%) who died [53 of 213 (24.9%) in house, and 28 of 213 (13.1%) after discharge. At baseline, there were no significant differences between the groups (mRS 0–2 vs mRS 3–6) regarding age, serum creatinine, vascular risk factors, stroke etiology, as well as frequency of systemic thrombolysis and/or mechanical thrombectomy.

### Univariate analysis of blood-based biomarkers at baseline and outcome after 3 months

**Figure 2.**
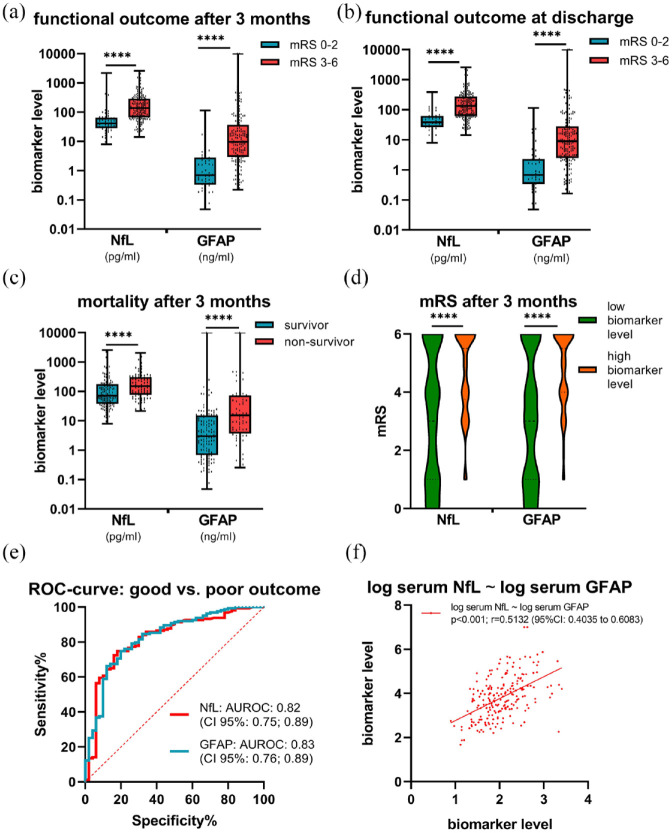
Serum biomarkers in patients with ischemic stroke. (a) Serum biomarkers according to 3-months mRS (mRS 0–2 vs mRS 3–6). (b) Serum biomarkers according the mRS at discharge (mRS 0–2 vs mRS 3–6). (c) Mortality at follow-up 3 months after index stroke. (d) mRS according to median biomarker levels (NfL: 96 pg/ml; GFAP: 5.7 ng/ml). (e) ROC-curve good versus poor outcome 3 months after index stroke. (f) Spearman’s correlations among biomarkers serum NfL ~ serum GFAP. *p-value < 0.05; **p-value < 0.01; ***p-value < 0.001; ****p-value < 0.0001.

Patients with poor functional outcome had significantly higher serum biomarker-levels [median NfL: 136 pg/ml (IQR, 67; 277); median GFAP: 9.6 ng/ml (IQR, 3; 33)] than those with good functional outcome [median NfL: 41 pg/ml (IQR, 29; 62), *p* < 0.0001; median GFAP: 0.7 ng/ml (IQR, 0.3; 2.7), *p* < 0.0001] (Figure 2(a)). Further, we explored the association between serum biomarkers and mortality at 3-month follow-up. Comparing the two groups, non-survivors [median NfL: 150 pg/ml (IQR, 76; 306); GFAP: 15.4 ng/ml (IQR, 3.7; 74)] showed increased serum NfL (*p* < 0.0001) and GFAP (*p* < 0.0001) levels compared to survivors [median NfL: 71 pg/ml (IQR, 38; 176); GFAP: 3.0 ng/ml (IQR, 0.7; 15)] (Supplemental Table S1, Figure 2(c)). By distinguishing patients according to the median serum levels of NfL (96 pg/ml) and GFAP (5.7 ng/ml) (Supplemental Table S2), patients with higher biomarker levels at baseline had higher mRS scores at 3-month follow-up [3 (95% CI: 1; 6) vs 5.5 (95% CI: 3.75; 6) with elevated NfL: *p* < 0.0001; 3 (95% CI: 1; 6)] vs 5.5 (95% CI: 4; 6) with elevated GFAP: *p* < 0.0001] (Figure 2(d)). Blood-based biomarkers were also significantly associated and predictive for short-term prognosis, which is shown in detail in the Supplemental Materials.

### Associations and regression models

We found significant correlations between functional outcome 3 months after the index stroke and serum biomarker levels of NfL [*p* < 0.0001; *r* = 0.47 (95% CI: 0.36–0.58)] and GFAP [*p* < 0.0001; *r* = 0.48 (95% CI: 0.37–0.58)]. In addition, we found significant correlations between serum NfL and GFAP levels [*p* < 0.0001; *r* = 0.51 (95% CI: 0.40–0.61)] (Figure 2(f)). Results of the unadjusted and adjusted regression models are shown in Table 2. After adjustment for the predictors age, NIHSS on admission, ASPECTS on admission (8–10vs 0–7), mechanical thrombectomy (yes/no), systemic lysis therapy (yes/no), and pre-stroke mRS (0–2vs 3–6) NfL [OR 2.6 (95% CI: 1.6; 4.6)] and GFAP [OR 2.2 (95% CI: 1.6; 3.1)] displayed an association with poor outcome.

**Table 2.** Logistic regression unadjusted and adjusted for patients’ age, NIHSS score at hospital admission, ASPECTS at hospital admission (0–7vs 8–10), systemic thrombolysis (yes/no), mechanical thrombectomy (yes/no) and pre-stroke mRS (0–2 vs 3–5).

	Odds ratio [95% CI], unadjusted	Odds ratio [95% CI], adjusted
log (NfL)	3.94 [2.53; 6.55]	2.63 [1.62; 4.56]
log (GFAP)	2.16 [1.71; 2.81]	2.16 [1.58; 3.09]

### Clinical predicting models (without use of blood-based biomarkers)

**Figure 3.**
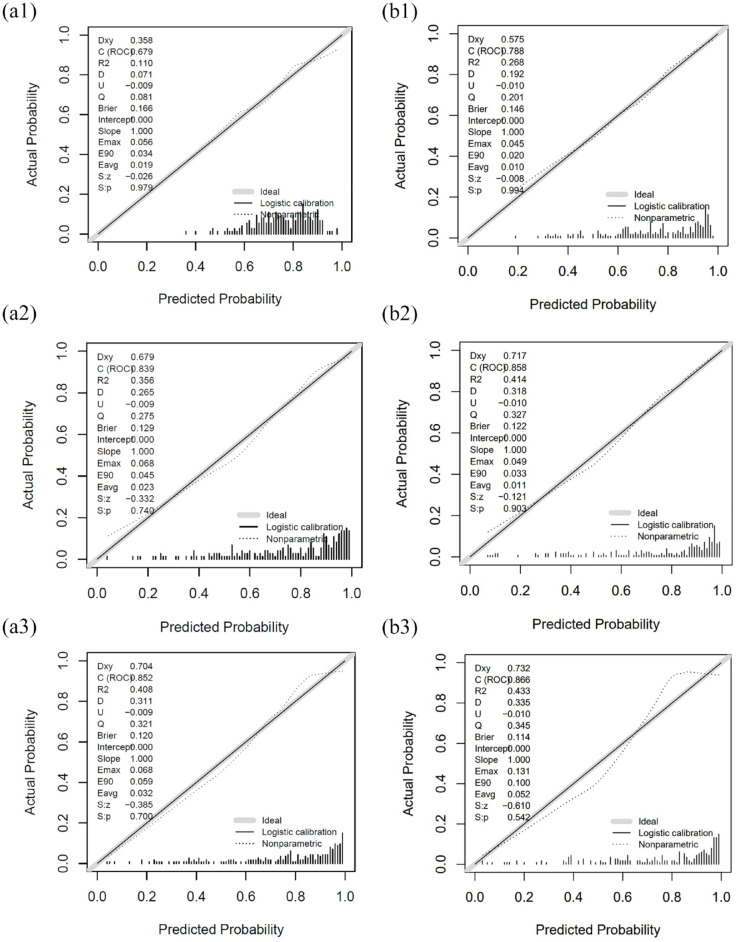
Calibration plots for model A and model B: Model A: mRS ~ age + NIHSS on admission. A1) (without biomarker). A2) + NfL. A3) + GFAP. Model B: mRS ~ age + NIHSS on admission + ASPECTS on admission (8–10 vs 0–7) + mechanical recanalization (yes/no). B1) (without biomarker). B2) + NfL. B3) + GFAP.

Results of three different models performed to evaluate associations between the biomarkers with the functional outcome after adjustment for potential confounders are shown in Tables 3 and 4. The calibration plots of model A and model B are shown in Figure 3. The calibration plots of model C are shown in the Supplemental Figure S2. The generated model A (mRS ~ age + NIHSS Score on admission) displayed an acceptable discrimination [AUROC 0.68 (95% CI: 0.61; 0.77)] and calibration. The overall performance was good [(Brier score 0.08 (95% CI: −0.07; 0.25)]. After inclusion of the parameters ASPECTS on admission and mechanical thrombectomy (yes/no) in model B [mRS ~ age + NIHSS on admission + ASPECTS on admission + mechanical thrombectomy (yes/no)], an improvement of discrimination [AUROC 0.79 (95% CI: 0.73; 0.86)], calibration and overall performance [Brier Score 0.19 (95% CI: 0.05; 0.36)] was shown compared with model A. Model C (using the parameter ASPECTS 24–72 h instead of ASPECTS on admission) also led to an improvement in discrimination [AUROC 0.73 (95% CI: 0.66; 0.82)], calibration and overall performance, compared to model A.

**Table 3.** Comparison of the discrimination (AUROC) of Model A, B, and C with and without the inclusion of blood based biomarkers.

AUROC	Without biomarkers	+NfL	*p-value*
Model A	0.68 [0.61; 0.77]	0.84 [0.78; 0.90]	*p* < *0.001*
Model B	0.79 [0.73; 0.86]	0.86 [0.80; 0.93]	*p = 0.029*
Model C	0.73 [0.66; 0.82]	0.85 [0.78; 0.92]	*p = 0.005*
AUROC	Without biomarkers	+GFAP	*p-value*
Model A	0.68 [0.61; 0.77]	0.85 [0.79; 0.91]	*p* < *0.001*
Model B	0.79 [0.73; 0.86]	0.87 [0.80; 0.93]	*p = 0.003*
Model C	0.73 [0.66; 0.82]	0.86 [0.78; 0.92]	*p = 0.001*
AUROC	Without biomarkers	+NfL and GFAP	*p-value*
Model A	0.68 [0.61; 0.77]	0.88 [0.83; 0.93]	*p* < *0.001*
Model B	0.79 [0.73; 0.86]	0.88 [0.85; 0.94]	*p* < *0.001*
Model C	0.73 [0.66; 0.82]	0.88 [0.84; 0.94]	*p* < *0.001*

**Table 4.** Calibration and overall performance of model A, B, and C.

	Without biomarkers	+NfL	+GFAP	+NfL and GFAP
Model A				
Emax	0.06 [0.04; 0.34]	0.07 [0.04; 0.24]	0.07 [0.04; 0.25]	0.08 [0.04; 0.21]
Eavg	0.02 [0.01; 0.05]	0.02 [0.01; 0.06]	0.03 [0.01; 0.07]	0.03 [0.01; 0.05]
Brier Score	0.08 [−0.07; 0.25]	0.28 [0.16; 0.47]	0.33 [0.19; 0.52]	0.38 [0.24; 0.57]
R2	0.11 [0.04; 0.24]	0.36 [0.23; 0.54]	0.41 [0.28; 0.58]	0.47 [0.35; 0.64]
Model B				
Emax	0.05 [0.03; 0.26]	0.05 [0.03; 0.25]	0.13 [0.06; 0.25]	0.12 [0.05; 0.23]
Eavg	0.01 [0.01; 0.05]	0.01 [0.01; 0.05]	0.05 [0.02; 0.08]	0.03 [0.01; 0.06]
Brier Score	0.19 [0.05; 0.36]	0.32 [0.19; 0.53]	0.37 [0.20; 0.56]	0.39 [0.27; 0.60]
R2	0.27 [0.16; 0.43]	0.41 [0.30; 0.60]	0.43 [0.29; 0.61]	0.48 [0.38; 0.66]
Model C				
Emax	0.03 [0.04; 0.29]	0.08 [0.04; 0.26]	0.08 [0.04; 0.21]	0.11 [0.04; 0.23]
Eavg	0.01 [0.01; 0.06]	0.03 [0.01; 0.06]	0.04 [0.01; 0.06]	0.03 [0.01; 0.06]
Brier Score	0.12 [−0.02; 0.32]	0.30 [0.17; 0.50]	0.32 [0.17; 0.52]	0.37 [0.24; 0.58]
R2	0.18 [0.09; 0.34]	0.38 [0.24; 0.57]	0.41 [0.27; 0.58]	0.47 [0.37; 0.64]

### Incremental value of serum NfL in predicting functional outcome

Following the inclusion of the blood-based biomarker serum NfL in model A, a significant enhancement in discrimination was observed [AUROC 0.84 (95% CI: 0.78; 0.90) vs 0.68 (95% CI: 0.61; 0.77); *p* < 0.001], along with improved overall performance [Brier score with additional use of NfL: 0.28 (0.16; 0.47) compared to without biomarkers: 0.08 (−0.07; 0.25)] in model A. However, calibration showed a slight decrease [Emax 0.28 (0.16; 0.47) and Eavg 0.01 (0.01; 0.05) vs without biomarkers: Emax 0.08 (−0.07; 0.25) and Eavg 0.11 (0.04; 0.24)]. In Model B discrimination showed a significant improvement [AUROC 0.86 (95% CI: 0.80; 0.93) vs 0.79 (95% CI: 0.73; 0.86); *p* = 0.029] and overall performance was enhanced [Brier Score with additional use of NfL: 0.32 (95% CI: 0.19–0.53) and without biomarker: 0.19 (95% CI: 0.05–0.36)]. Calibration showed no difference with and without use of serum NfL. Likewise, in Model C, the addition of serum NfL significantly improved discrimination [AUROC 0.85 (95% CI: 0.78; 0.92) vs 0.73 (95% CI: 0.66; 0.82); *p* = 0.005] and enhanced calibration [Brier Score with additional use of NfL: 0.30 (95% CI: 0.17; 0.50) and without biomarkers: 0.12 (95% CI: −0.02; 0.32)], but calibration was marginally worse.

### Incremental value of serum GFAP in predicting functional outcome

Including serum GFAP demonstrated a significant enhancement in predictive power, as evidenced by an improved discrimination [AUROC 0.85 (95% CI: 0.79; 0.91) vs 0.68 (95% CI: 0.61; 0.77); *p* < 0.001] and an overall performance enhancement [Brier score with the addition of GFAP: 0.33 (95% CI: 0.19; 0.52) and without the biomarker: 0.08 (95% CI: −0.07; 0.25)] when integrated into model A. However, it’s important to note that the calibration exhibited a slight decrease [Emax: 0.07 (0.04; 0.25) and Eavg 0.03 (0.01; 0.07)]. In model B with the supplementary use of GFAP, discrimination was achieved with an AUROC of 0.87 (95% CI: 0.80; 0.93). In comparison to Model B without the biomarkers, the improvement in discrimination was statistically significant (*p* = 0.003), and calibration was enhanced [Brier Score with the addition of GFAP: 0.37 (0.20; 0.56) and without the biomarker: 0.19 (95% CI: 0.05; 0.36)], albeit with a marginal decrease in calibration. Model C demonstrates promising results, albeit with a slight performance decrease compared to Model B.

### Incremental value of both serum biomarkers in predicting functional outcome

Upon integration of both serum biomarkers, a significant enhancement in predictive capacity was observed, as evidenced by improved discrimination [AUROC 0.88 (95% CI: 0.83; 0.93) vs 0.68 (95% CI: 0.61; 0.77); *p* < 0.001] and overall performance [Brier score with both serum biomarkers: 0.37 (0.24; 0.58) and without the biomarkers: 0.08 (95% CI: −0.07; 0.25)] within model A compared to without biomarkers. Again, the calibration showed a slight decrease [Emax 0.08 (0.04; 0.21) and Eavg 0.08 (0.04; 0.21)]. Model B with the use of both biomarkers displayed the highest discrimination among the three prognostic models, with an AUROC of 0.88 (95% CI: 0.85; 0.94). Compared to Model B without biomarkers, this improvement in discrimination was statistically significant (*p* < 0.001), and overall performance was enhanced [Brier Score with both biomarkers: 0.39 (95% CI: 0.27; 0.60) and without biomarkers: 0.19 (95% CI: 0.05; 0.36)], although with a marginal decrease in calibration. Similarly, in Model C, the inclusion of both serum biomarkers significantly improved discrimination [AUROC 0.88 (95% CI: 0.84; 0.94) vs 0.734 (95% CI: 0.66; 0.82); *p* = 0.001] and enhanced the overall performance [Brier Score with both serum biomarkers: 0.37 (95% CI: 0.24; 0.58) and without the biomarker: 0.12 (−0.02; 0.32)], although calibration showed a marginal decrease. The comparison between the biomarkers (NfL vs GFAP vs NfL + GFAP) within the models did not yield any significant differences in the discrimination, calibration or overall performance.

## Discussion

Personalized outcome prediction after severe acute ischemic stroke is highly relevant for the extent of rehabilitation and for the communication to patients and relatives in the acute phase of stroke. With such a personalized prediction also the opportunity arises for a better comparison of different therapeutic strategies. Recent studies showed an association of the functional outcome after ischemic stroke and the two blood-based biomarkers NfL and GFAP.^5,6,8,20–25^ However, predictive models incorporating these two biomarkers for short- and long-term functional outcome have not been implemented in practice yet. In this study we report the incremental value of serum NfL and GFAP in predicting the functional outcome in patients with severe acute ischemic stroke of the anterior circulation.

### Blood-based biomarkers and 3-months functional outcome

Our data reveal a significant correlation between serum levels of NfL and GFAP and functional outcomes 3 months after index stroke. Furthermore, the observed association of these two biomarkers with death within 3 months after index stroke highlights their potential as indicators for poor or limiting prognosis and might be helpful in deciding to continue treatment or change therapeutic goals. In previous publications, serum NfL has been established as an indicator for minor strokes and microangiopathy.^7,26,27^ In patients with severe ischemic stroke, previous reports suggest a significant role of blood NfL and GFAP in prognosticating functional outcomes and are in line with our findings.^5,6,20–25^ However, key limitation of these prior studies were primary the study methodologies and the small sample sizes, resulting in restricted testing as an increase of risk of drawing a false-positive conclusion and limitations in generalizability.^5,6,21–24^ Although these studies showed significant associations of serum NfL and GFAP with outcome, the incremental prognostic value of these two biomarkers has not been determined yet.

### Incremental value of blood-based biomarkers to known determinants

In the context of predicting the long-term outcome, it becomes imperative to assess the added significance of novel determinants like blood-based biomarkers. In case of predicting the functional outcome, which is characterized by a binary classification (good vs poor outcome), discrimination may carry more weight compared to overall performance and calibration.^16,28^ Adding serum NfL and/or GFAP resulted in a significant enhancement in the prognostic accuracy of all models measured by comparison of AUROC. This improvement underscores the prognostic value, these biomarkers provide beyond the clinical and radiographic factors previously established. The best AUROC was achieved by including the determinants age, NIHSS on admission, ASPECTS on admission, mechanical thrombectomy (yes/no) and both serum biomarkers. In our analysis both the Brier Score and R2 demonstrated enhancements, indicating a refined predictive ability of the models. However, it’s important to note that despite achievements in discrimination (AUROC) and overall performance (Brier Score and R2), there was a decline in calibration (Emax and Eavg) in all three models after addition of the biomarkers.

Remarkably, with the integration of serum NfL and GFAP, every model experienced a significant improvement in their discriminative capabilities and overall performance, ultimately converging toward consistently robust AUROC values. This underscores the crucial role blood-based biomarkers might play in enhancing prognostication in acute severe ischemic stroke. Even with the inclusion of just two established determinants, age and NIHSS on admission, we observed a substantial improvement in discrimination after incorporating serum NfL and/or GFAP. Specifically, the addition of serum NfL elevated the discrimination to an impressive 0.84, while serum GFAP improved it to 0.85 within the model. Also the addition of both biomarkers to the models yielded numerically higher discrimination; the difference to the models with only one biomarker was not statistical significant.

### Optimal utilization at standardized points in time in each blood-based biomarker

When comparing serum NfL and GFAP within each model, it appears that serum GFAP may lead in terms of discrimination and overall performance. However, it looks like the biomarkers are equivalent in their prognostic value and that there is no significant benefit from using both biomarkers. Therefore, the choice of biomarker should be based on factors such as cost and the availability of a reliable measurement method, which currently favors serum NfL. In case of predicting short-term outcomes (supplement), both serum NfL and GFAP demonstrate associations with early clinical and radiological measures of stroke severity. However, previous publications have shown that serum NfL tends to increase over the first few days to weeks, in contrast to GFAP, which reaches its peak after approximately 24 h.^7,29^ Consequently, if it is feasible to assess both biomarkers, an option could be to measure serum GFAP standardized in the acute phase of stroke (such as on day 1 after index stroke) and serum NfL on day 3 respectively 7 after index stroke due to its robustness.

To the best of our knowledge, this study is the first to demonstrate the incremental prognostic value of serum NfL and GFAP in patients with severe ischemic stroke for functional outcome 3 months after index stroke. Notably, our study is characterized by its unselected, large longitudinal prospective design, involving a cohort encompassing 213 patients diagnosed with severe acute ischemic stroke. However, it’s imperative to approach the introduction of new variables into predictive models with caution. Data validation and thorough model evaluation are essential steps to verify that the newly added variables truly enhance the model’s predictive capabilities, without introducing noise or undue complexity. In earlier publications, the association between NfL and functional outcomes has been less clear.^30^ One possible explanation is that previous studies included stroke patients across a wide spectrum of clinical severity. Our present analysis targets specifically patients with severe acute ischemic stroke exclusively in the anterior circulation territory. By this selection we excluded stroke patients with, for instance, minor strokes or brainstem ischemia, where a small lesion can lead to a severe functional outcome. A potentially smaller effect can be anticipated due to comorbidities such as pre-existing microangiopathy or heart failure^7,31^ resulting in an increased baseline level of serum NfL/GFAP. This circumstance makes it more challenging to differentiate between the amount of elevation of serum NfL/GFAP induced by the comorbidities and by minor stroke.

### Strengths and limitations

The main strengths of our study are the prospective design and the detailed statistical analysis studying discrimination, calibration and overall performance on basis of a study cohort of 213 unselected stroke patients. These conditions built the ground for developing three valid prognostic models to assess the incremental value of serum NfL and GFAP levels additive to known determinants. Data collection was done blinded to follow-up and biomarker measurement. In addition, our selection criteria included only patients with NIHSS ⩾ 6 on admission or indication for mechanical thrombectomy, which allowed us to explore the prognostic value of blood-based biomarkers in severe stroke patients.

Our study has limitations. First, blood-based biosamples were measured at a single point in time, while serum levels of NfL and GFAP are known to gradually increase hours after symptom onset and reaching maximum plasma levels at different timepoints days after stroke onset.^7,29^ Biomarker trajectories created from repeated measurements may contain additional information regarding the development of the short- and long-term functional outcome. However, although outcome prognostication could be helpful in the early hours of stroke concerning the indication and selection of acute stroke therapies, biomarkers obtained beyond 48 h might be helpful in deciding to continue treatment or change therapeutic goals.^7,29^ Second, plasma biomarkers levels before stroke onset were unknown. A history of neurological diseases prior to ischemic stroke may have affected the patients’ biomarker levels. Thirdly, we chose the ASPECT Score over infarct volume due to practical limitations. Although each ASPECTS value is inherently tied to different infarct volumes and shows a robust correlation with functional outcome,^32^ it’s essential to recognize that this correlation may not universally apply to smaller infarct volumes. Another limitation relates to the consideration of the ASPECTS and pmRS score as dichotomous instead of linear variables. This may have resulted in a loss of information and a distortion of the observed results. A fifth limitation of our study is still the relatively small sample size of 213 patients with severe ischemic stroke. To ensure the robustness and generalizability of our findings, further research is required with larger patient populations. Validation of our results through both internal and external replication in independent cohorts is imperative.

## Conclusion

In conclusion, this is the first study demonstrating the potential incremental prognostic value of serum NfL and GFAP predicting good functional outcome 3 months after severe ischemic stroke. Prediction of functional outcome after severe ischemic stroke was more accurate using the blood-based biomarkers NfL and GFAP. These findings need to be replicated in independent external cohorts before its role in personalized outcome prediction can be judged.

## Non-standard abbreviations and acronyms

ASPECTS Alberta Stroke Program CT Score

GFAP Glial fibrillary acidic protein

mRS modified Rankin Scale

NfL Neurofilament light chain protein

NIHSS National Institutes of Health Stroke Scale

ΔNIHSS NIHSS change (NIHSS 24 h after admission - baseline NIHSS)

## Supplemental Material

sj-docx-1-eso-10.1177_23969873241234436 – Supplemental material for Incremental value of serum neurofilament light chain and glial fibrillary acidic protein as blood-based biomarkers for predicting functional outcome in severe acute ischemic stroke Supplemental material, sj-docx-1-eso-10.1177_23969873241234436 for Incremental value of serum neurofilament light chain and glial fibrillary acidic protein as blood-based biomarkers for predicting functional outcome in severe acute ischemic stroke by Christoph Vollmuth, Cornelia Fiessler, Felipe A Montellano, Alexander M Kollikowski, Fabian Essig, Patrick Oeckl, Lorenzo Barba, Petra Steinacker, Cara Schulz, Kathrin Ungethüm, Judith Wolf, Mirko Pham, Michael K Schuhmann, Peter U Heuschmann, Karl Georg Haeusler, Guido Stoll, Markus Otto and Hermann Neugebauer in European Stroke Journal
