# Supplementary material for: Incremental value of serum neurofilament light chain and glial fibrillary acidic protein as blood-based biomarkers for predicting functional outcome in severe acute ischemic stroke
Source: Eur Stroke J. 2024 Feb 24;9(3):751–62. doi: 10.1177/23969873241234436 (PMC11418447; doi:10.1177/23969873241234436)
Supplement: sj-docx-1-eso-10.1177_23969873241234436 – Supplemental material for Incremental value of serum neurofilament light chain and glial fibrillary acidic protein as blood-based biomarkers for predicting functional outcome in severe acute ischemic stroke [file sj-docx-1-eso-10.1177_23969873241234436.docx]

**SUPPLEMENTAL MATERIAL**

**Incremental value of serum neurofilament light chain and glial fibrillary acidic protein as blood-based biomarkers for predicting functional outcome in severe acute ischemic stroke**

**RESULTS**

*Associations of blood-based biomarkers and baseline characteristics*

Both serum biomarker levels were not associated with sex (NfL: p=0.29; GFAP: p=0.30). There was no difference between the groups in terms of acute treatment (systemic lysis, mechanical thrombectomy). In addition, etiology of stroke was not associated with serum NfL or GFAP level.

*Associations of blood-based biomarkers regarding high and low biomarker level*

We distinguished patients according to the median serum levels of each biomarker (NfL: 96pg/ml; GFAP: 5.7ng/ml) (**Supplementary Table S2**). We observed that patients with high serum biomarker levels showed higher NIHSS scores on admission (NfL: p=0.0114; GFAP: p=0.0004), at day 1 (NfL: p<0.0001; GFAP: p<0.0001), day 2 (NfL: p<0.0001; GFAP: p<0.0001) and day 3 (NfL: p<0.0001; GFAP: p<0.0001) and at discharge (NfL: p<0.0001; GFAP: p<0.0001) compared to patients with lower biomarkers concentrations (**Supplement Figure S1C-1D and Supplement Table S2)**. Furthermore, the mean creatinine levels was higher in patients with high serum NfL level (NfL: p=0.0018), while there was no difference between mean creatinine and serum GFAP level (p=0.065). In patients with high serum NfL and GFAP levels blood sampling was performed significantly later than in patients with low serum NfL (p<0.0001) and GFAP (p=0.0004) levels.

*Associations of blood-based biomarkers regarding ASPECTS on admission*

By distinguishing patients into two categories according to the admission ASPECTS, patients with ASPECTS < 8 disclosed increased NfL (p<0.0001) and GFAP (p<0.0001) levels compared to patients with ASPECTS ≥ 8 (**Supplement Figure S 1A** and **Supplementary Table S3**). Similar, patients with ASPECTS < 8 measured 24-72h after index stroke disclosed increased NfL (p=0.0005) and GFAP (p<0.0001) levels compared to patients with ASPECTS ≥ 8 (**Supplement Figure S1B**).

*Correlations of blood-based biomarkers and short-term outcome*

There was a significant correlation between baseline NIHSS and serum levels of NfL [p=0.019; r=0.1614 (95%CI: 0.02325 to 0.2935)] as well as GFAP [p<0.0001; r=0.3087 (95%CI: 0.1777 to 0.4290)] (**Supplement Figure S1E**). Further, we found a correlation between time from stroke onset to mechanical thrombectomy and serum GFAP level [p=0.023; r=0.218 (95%CI: 0.02509 to 0.3960)], while there was no correlation between serum NfL and time between clinical onset to mechanical thrombectomy (p=0.26). The admission ASPECTS was significantly associated with serum levels of NfL [p<0.0001; r=-0.2648 (95%CI: -0.3895 to -0.1306)] and GFAP [p<0.0001; r=-0.5924 (95%CI: -0.6758 to -0.4941). Similar, the ASPECTS 24-72 after stroke onset was significantly associated with serum level of NfL [p<0.0001; r=-0.3571 (95%CI: -0.4742 to -0.2275)] and GFAP [p<0.0001; r=-0.6924 (95%CI: -0.7593 to -0.6110).

*Associations and regression models*

Furthermore, we incorporated the NIHSS Score at the time of blood sampling, as opposed to the score upon admission, into our new models. When compared to regression models utilizing NIHSS Score on admission, the impact of blood-based biomarkers on functional outcomes is only marginally reduced here, yet remains statistically significant. The results of adjusted regression models are presented in **Supplementary Table S4**. Upon adjusting for predictors age, NIHSS at the time of blood sampling, ASPECTS on admission (8-10 vs. 0-7), and mechanical thrombectomy (yes/no), both NfL [OR 2.95 (95% CI: 1.71; 5.62)] and GFAP [OR 1.57 (95% CI: 1.16; 2.18)] demonstrated an association with poor outcome. Notably, a robust association with poor outcomes was also achieved after adjustment for only age and NIHSS at the time of blood sampling, with NfL [OR 3.28 (95% CI: 1.91; 6.22)] and GFAP [OR 1.71 (95% CI: 1.30; 2.33)].

*Incremental value of blood-based biomarkers (NfL, GFAP, NfL+GFAP) to known determinants (functional outcome mRS 0-4 vs. 5-6)*

Additionally, we introduced Models A, B, and C, distinguishing between a good functional outcome (mRS 0-4) and a poor functional outcome (mRS 5-6) (refer to **Supplementary Table S5**). In terms of discrimination, these models exhibited remarkably similar AUROC values compared to the models that categorized functional outcomes as mRS 0-2 vs. 3-6. The incorporation of blood-based biomarkers into the prediction of mRS 5-6 also led to a significant enhancement in discrimination.

**DISCUSSION**

As a secondary finding, our study provides further evidence regarding the potential utility of serum biomarkers in predicting prognosis during the acute phase of ischemic stroke. In detail, levels of the two blood-based biomarker are associated with early clinical and radiological measures of stroke severity. In alignment with previous research, we corroborate an association between serum NfL as well as serum GFAP concentration and the admission ASPECTS [1-3], suggesting a direct relationship between those two biomarker levels and the extent of the ischemic injury. Equally, there was a significant association between serum NfL and GFAP concentration and the ASPECTS assessed 24-72 hours after stroke onset, confirming those two biomarkers as a surrogate of the extent of brain injury. Most interestingly, the associations between serum NfL and GFAP and clinical measures in the time-course suggest a possible role of these biomarkers in monitoring patients at risk.

|  | | | **Supplementary Table S1** | **survivors (n=132)** | **non-survivors (n=81)** | **p-value *** |
| --- | --- | --- | --- | --- | --- | --- |
| baseline data | | | |  |  |  |
|  | mean age, years (±SD) | | | 72.9 (± 12.8) | 81.4 (± 9.9) | **<0.0001** |
|  | sex, female, n (%) | | | 63 (47.7) | 37 (45.7) | 0.78 |
|  | pre stroke mRS 0-2, n (%) | | | 14 (10.6) | 22 (27.2) | **0.002** |
|  | wake-up stroke, n (%) | | | 39 (29.5) | 30 (37.0) | 0.29 |
|  | mean creatinin, mg/dl (±SD) | | | 1.06 (±0.6) | 1.21 (±0.7) | **0.005** |
|  | mean GFR, ml/min/173cm^2^ (±SD) | | | 77.5 [57; 94] | 61 [45; 81] | **0.0003** |
|  | time from onset to mechanical recanalisation, hours^a,b^, mean (±SD) | | | 4.1 (±3.1) | 4.6 (±3.3) | 0.42 |
| vascular risk factors, n (%) | | | |  |  |  |
|  | hypertension | | | 90 (68.2) | 55 (67.9) | >0.99 |
|  | diabetes mellitus | | | 27 (20.5) | 15 (18.5) | 0.86 |
|  | heart failure | | | 12 (9.1) | 19 (23.5) | **0.005** |
|  | atrial fibrillation | | | 55 (41.7) | 44 (54.3) | 0.09 |
| stroke severity^c^, median [IQR] | | | |  |  |  |
|  | NIHSS on admission | | | 11 [8; 15] | 15 [11; 18] | **<0.0001** |
|  | NIHSS at 24h | | | 7 [3; 15] | 19 [12; 29] | **<0.0001** |
|  | NIHSS at 72h | | | 5 [2; 13] | 15 [11; 24] | **<0.0001** |
|  | NIHSS at discharge | | | 5 [1; 12] | / | **/** |
| etiology, n (%) | | | |  |  |  |
|  | large artery atherosclerosis | | | 23 (17.4) | 3 (3.7) | **0.004** |
|  | cardioembolism | | | 59 (44.7) | 39 (48.1) | 0.67 |
|  | unknown/others | | | 50 (37.9) | 39 (48.1) | 0.15 |
| neuoradiologic data | | | |  |  |  |
|  | blood vessel occluded, n (%) | | |  |  |  |
|  |  | | ICA | 32 (24.2) | 16 (19.8) | 0.50 |
|  |  | | M1 | 56 (42.4) | 43 (53.1) | 0.16 |
|  |  | | M2 | 32 (24.2) | 16 (19.8) | 0.50 |
|  | ASPECTS, median [IQR] | | |  |  |  |
|  |  | | ASPECTS on admission | 8 [6.5; 9] | 7 [6; 8] | **0.004** |
|  |  | | ASPECTS 24-72h | 7 [6; 8] | 5.5 [2; 7] | **<0.0001** |
|  | collateral status, n (%) | | |  |  |  |
|  |  | | 1 | 53 (40.1) | 42 (51.9) | 0.12 |
|  |  | | 2 | 32 (24.2) | 24 (29.6) | 0.42 |
|  |  | | 3 | 24 (18.1) | 8 (9.9) | 0.12 |
|  |  | | not assessable | 23 (17.4) | 7 (8.6) | 0.10 |
| acute treatment, n (%) | | | |  |  |  |
|  | systemic lysis | | | 57 (43) | 28 (34) | 0.25 |
|  | mechanical recanalisation | | | 94 (71) | 64 (79) | 0.26 |
|  | TICI ≥ 2b ^d^ | | | 86 (91) | 46 (56) | **0.014** |
| biomarker data, median [IQR] | | | |  |  |  |
|  | time from admission to blood sample, days | | | 1 [1; 2.75] | 1 [1; 2] | 0.35 |
|  | NfL, pg/ml | | | 71 [38; 176] | 150 [76; 306] | **<0.0001** |
|  | GFAP, ng/ml | | | 2.9 [0.7; 15] | 15 [3.7; 74] | **<0.0001** |
|  | | a: only patients with clear time windows | | | | |
|  | | b: only patients with mechanical recanalisation | | | | |
|  | | c: deceased patients excluded | | | | |
|  | | d: TICI Score from patients with mechanical recanalisation | | | | |

| **Supplementary Table S2** | | | **NfL (cut-off: 96pg/ml)** | | | **GFAP (cut-off: 5.7ng/ml)** | | |
| --- | --- | --- | --- | --- | --- | --- | --- | --- |
|  | |  | low biomarker level (n=107) | high biomarker level (n=106) | p-value | low biomarker level (n=107) | high biomarker level (n=106) | p-value |
| baseline data | | |  |  |  |  |  |  |
|  | mean age, years (±SD) | | 75.4 (±12.5) | 76.9 (±12.4) | 0.24 | 77.4 (±11.5) | 74.9 (±13.3) | 0.31 |
|  | sex, female, n (%) | | 51 (47.7) | 49 (46.2) | 0.84 | 51 (47.7) | 49 (46.2) | 0.84 |
|  | pre stroke mRS 0-2, n (%) | | 95 (88.8) | 82 (77.4) | **0.027** | 89 (82.2) | 88 (83.0) | 0.98 |
|  | wake-up stroke, n (%) | | 33 (30.8) | 35 (33.0) | 0.73 | 34 (31.8) | 34 (32.1) | 0.96 |
|  | mean creatinin, mg/dl (±SD) | | 1.0 (±0.3) | 1.3 (±0.9) | **0.0018** | 1.17 (±0.69) | 1.06 (±0.63) | 0.065 |
|  | mean GFR, ml/min/173cm^2^ (±SD) | | 77.7 (±25.8) | 65.2 (±27.5) | **0.0012** | 67.3 (±25.5) | 75.7 (±28.6) | **0.047** |
|  | time from onset to mechanical recanalisation, hours^a,b^, mean (±SD) | | 4.2 (±2.8) | 4.4 (±3.5) | 0.55 | 3.8 (±2.5) | 4.8 (±3.6) | **0.029** |
| vascular risk factors, n (%) | | |  |  |  |  |  |  |
|  | hypertension | | 66 (61.7) | 79 (74.5) | **0.045** | 75 (70.1) | 70 (66.0) | 0.53 |
|  | diabetes mellitus | | 16 (15.0) | 26 (24.5) | 0.08 | 21 (19.6) | 21 (19.8) | 0.97 |
|  | heart failure | | 16 (15.0) | 15 (14.2) | 0.87 | 52 (48.6) | 47 (44.3) | 0.54 |
|  | atrial fibrillation | | 50 (46.7) | 49 (46.2) | 0.94 | 17 (15.9) | 14 (13.2) | 0.58 |
| stroke severity^c^, median [IQR] | | |  |  |  |  |  |  |
|  | NIHSS at admission | | 12 [8; 16] | 14 [10; 18] | **0.0114** | 11 [8; 15] | 15 [10; 18] | **0.0004** |
|  | NIHSS at 24h | | 7 [3; 14] | 18 [8; 27] | **<0.0001** | 6 [3; 13] | 18 [11; 26] | **<0.0001** |
|  | NIHSS at 48h | | 6 [2; 14] | 16 [7; 28] | **<0.0001** | 5 [2; 13] | 16 [10; 25] | **<0.0001** |
|  | NIHSS at 72h | | 5 [2; 12] | 14 [7; 21] | **<0.0001** | 4 [1; 11] | 14 [10; 23] | **<0.0001** |
|  | NIHSS at discharge | | 3 [1; 9] | 10 [3; 15] | **<0.0001** | 2 [1; 5] | 11 [6; 15] | **<0.0001** |
|  | mRS at discharge | | 4 [2; 5] | 5 [4; 6] | **<0.0001** | 3 [2; 5] | 5 [4; 6] | **<0.0001** |
|  | mRS at follow up | | 3 [1; 6] | 5.5 [3.75; 6] | **<0.0001** | 3 [1; 6] | 5.5 [4; 6] | **<0.0001** |
| etiology, n (%) | | |  |  |  |  |  |  |
|  | large artery atherosclerosis | | 13 (12.1) | 13 (12.3) | 0.98 | 11 (10.3) | 15 (14.2) | 0.40 |
|  | cardioembolism | | 48 (44.9) | 50 (47.2) | 0.74 | 52 (49.1) | 46 (43.4) | 0.45 |
|  | unknown/others | | 46 (43.0) | 43 (40.6) | 0.72 | 42 (39.2) | 44 (41.5) | 0.74 |
| neuoradiologic data | | |  |  |  |  |  |  |
|  | blood vessel occluded, n (%) | |  |  |  |  |  |  |
|  |  | ICA | 20 (18.7) | 30 (28.3) | 0.09 | 21 (19.6) | 29 (27.4) | 0.18 |
|  |  | M1 | 53 (49.6) | 48 (45.3) | 0.54 | 48 (44.9) | 53 (50.0) | 0.45 |
|  |  | M2 | 23 (21.5) | 27 (25.5) | 0.49 | 30 (28.0) | 20 (18.9) | 0.11 |
|  | ASPECTS, median [IQR] | |  |  |  |  |  |  |
|  |  | ASPECTS on admission | 8 [7; 9] | 7 [6; 8] | **0.0003** | 8 [7; 9] | 7 [5; 8] | **<0.0001** |
|  |  | ASPECTS 24-72h | 7 [6; 8.25] | 6 [2; 7] | **<0.0001** | 8 [7; 9] | 5 [2; 7] | **<0.0001** |
|  | collateral status, n (%) | |  |  |  |  |  |  |
|  |  | 1 | 44 (41.1) | 51 (48.1) | 0.38 | 39 (36.4) | 56 (52.8) | **0.027** |
|  |  | 2 | 28 (26.2) | 28 (26.4) | 0.99 | 32 (29.9) | 24 (22.6) | 0.20 |
|  |  | 3 | 19 (17.8) | 13 (12.3) | 0.25 | 19 (17.8) | 13 (12.3) | 0.24 |
|  |  | not assessable | 16 (15.0) | 14 (13.2) | / | 17 (15.9) | 13 (12.3) | / |
| acute treatment, n (%) | | |  |  |  |  |  |  |
|  | systemic lysis | | 44 (41.1) | 41 (38.7) | 0.72 | 47 (43.9) | 38 (35.8) | 0.23 |
|  | mechanical recanalisation | | 79 (73.8) | 79 (74.5) | 0.91 | 77 (72.0) | 81 (76.4) | 0.46 |
|  | TICI ≥ 2b ^d^ | | 68 (86.1) | 64 (81.0) | 0.64 | 69 (89.6) | 63 (77.8) | 0.09 |
| biomarker data, median [IQR] | | |  |  |  |  |  |  |
|  | time from admission to blood sample, days | | 1 [1; 2] | 1 [1; 3] | **<0.0001** | 1 [1; 2] | 1 [1; 3] | **0.0004** |
|  | NfL, pg/ml | | 51 [31; 67] | 228 [144; 380] | **<0.0001** | 62 [35; 125] | 173 [72; 338] | **<0.0001** |
|  | GFAP, ng/ml | | 2.3 [0.5; 9] | 16 [4; 75] | **<0.0001** | 1.6 [0.5; 3.0] | 22 [12; 83] | **<0.0001** |
|  | a: only patients with clear time windows | | | | | | |  |
|  | b: only patients with mechanical recanalisation | | | | | | |  |
|  | c: deceased patients excluded | | | | | | |  |
|  | d: TICI Score from patients with mechanical recanalisation | | | | | |  |  |

|  | | | **Supplementary Table S3** | **ASPECTS ≥ 8 (n=105)** | **ASPECTS < 8 (n=108)** | **p-value *** |  |
| --- | --- | --- | --- | --- | --- | --- | --- |
| baseline data | | | |  |  |  |  |
|  | mean age, years (±SD) | | | 78.1 (±10.2) | 74.3 (±14.1) | 0.07 |  |
|  | sex, female, n (%) | | | 48 (45.7) | 52 (48.1) | 0.46 |  |
|  | pre stroke mRS 0-2, n (%) | | | 88 (83.8) | 89 (81.4) | 0.79 |  |
|  | wake-up stroke, n (%) | | | 27 (25.7) | 42 (38.9) | **0.041** |  |
|  | mean creatinin, mg/dl (±SD) | | | 1.2 (±0.7) | 1.0 (±0.6) | **0.0004** |  |
|  | mean GFR, ml/min/173cm^2^ (±SD) | | | 64 [45; 84] | 78.5 [58; 95.75] | **0.0007** |  |
|  | time from onset to mechanical recanalisation, hours^a,b^, hours, mean (±SD) | | | 4.0 (±3.2) | 4.7 (±3.0) | **0.029** |  |
| vascular risk factors, n (%) | | | |  |  |  |  |
|  | hypertension | | | 73 (69.5) | 72 (66.7) | 0.77 |  |
|  | diabetes mellitus | | | 22 (21.0) | 20 (18.5) | 0.66 |  |
|  | heart failure | | | 16 (15.2) | 15 (13.9) | 0.78 |  |
|  | atrial fibrillation | | | 55 (52.4) | 44 (40.7) | 0.09 |  |
| stroke severity^c^, median [IQR] | | | |  |  |  |  |
|  | NIHSS on admission | | | 15 [11; 18] | 10 [8; 15] | **<0.0001** |  |
|  | NIHSS at 24h | | | 6 [3; 15] | 16 [10; 23] | **<0.0001** |  |
|  | NIHSS at 72h | | | 4 [1; 11] | 14 [7; 20.75] | **<0.0001** |  |
|  | NIHSS at discharge | | | 2 [1; 6] | 10.5 [5; 15] | **<0.0001** |  |
|  | mRS at discharge | | | 3 [2; 5] | 5 [4; 6] | **<0.0001** |  |
|  | mRS at 3-month-FU | | | 3 [1; 6] | 5 [4; 6] | **<0.0001** |  |
| etiology, n (%) | | | |  |  |  |  |
|  | large artery atherosclerosis | | | 15 (14.3) | 11 (10.2) | 0.40 |  |
|  | cardioembolism | | | 50 (47.6) | 48 (44.4) | 0.78 |  |
|  | unknown/others | | | 40 (38.1) | 49 (45.4) | 0.21 |  |
| neuoradiologic data | | | |  |  |  |  |
|  | blood vessel occluded, n (%) | | |  |  |  |  |
|  |  | | ICA | 17 (16.2) | 31 (28.7) | **0.029** |  |
|  |  | | M1 | 44 (41.9) | 55 (50.9) | 0.19 |  |
|  |  | | M2 | 30 (28.6) | 18 (16.7) | **0.038** |  |
|  | collateral status, n (%) | | |  |  |  |  |
|  |  | | 1 | 32 (30.5) | 63 (58.3) | **<0.0001** |  |
|  |  | | 2 | 31 (29.5) | 25 (23.1) | 0.42 |  |
|  |  | | 3 | 23 (21.9) | 9 (8.3) | **0.0096** |  |
|  |  | | not assessable | 19 (18.1) | 11 (10.2) | 0.14 |  |
| acute treatment, n (%) | | | |  |  |  |  |
|  | systemic lysis | | | 49 (46.7) | 36 (33.3) | **0.048** |  |
|  | mechanical recanalisation | | | 81 (77.1) | 77 (71.3) | 0.33 |  |
|  | TICI ≥ 2b ^d^ | | | 67 (63.8) | 65 (60.2) | >0.99 |  |
| biomarker data, median [IQR] | | | |  |  |  |  |
|  | time from admission to blood sample, days | | | 1 [1; 2] | 1 [1; 3] | **0.033** |  |
|  | NfL, pg/ml | | | 69 [38; 154] | 137 [65; 302] | **<0.0001** |  |
|  | GFAP, ng/ml | | | 2.1 [0.5; 7.5] | 15.9 [4.8; 74.4] | **<0.0001** |  |
|  | | a: only patients with clear time windows | | | | | |
|  | | b: only patients with mechanical recanalisation | | | | | |
|  | | c: deceased patients excluded | | | | | |
|  | | d: TICI Score from patients with mechanical recanalisation | | | | |  |

**Supplementary Table S4**

Model A: Logistic regression adjusted for patients´ age and NIHSS Score.

|  | Odds Ratio [95% CI]  (NIHSS Score on admission) | Odds Ratio [95% CI]  (NIHSS Score at time of blood sampling) |
| --- | --- | --- |
| log (NfL) | 3.70 [2.34; 6.31] | 3.28 [1.91; 6.22] |
| log (GFAP) | 2.24 [1.73; 3.02] | 1.71 [1.30; 2.33] |

Model B: Logistic regression adjusted for patients´ age, NIHSS Score, ASPECTS at hospital admission (0-7 vs. 8-10) and mechanical thrombectomy (yes/no).

|  | Odds Ratio [95% CI]  (NIHSS Score on admission) | Odds Ratio [95% CI]  (NIHSS Score at time of blood sampling) |
| --- | --- | --- |
| log (NfL) | 3.08 [1.92; 5.29] | 2.95 [1.71; 5.62] |
| log (GFAP) | 2.01 [1.53; 2.75] | 1.57 [1.16; 2.18] |

Model C: Logistic regression adjusted for patients´ age, NIHSS Score, ASPECTS 24-72 hours (0-7 vs. 8-10) and mechanical thrombectomy (yes/no).

|  | Odds Ratio [95% CI]  (NIHSS Score on admission) | Odds Ratio [95% CI]  (NIHSS Score at time of blood sampling) |
| --- | --- | --- |
| log (NfL) | 3.50 [2.16; 6.09] | 3.21 [1.85; 6.16] |
| log (GFAP) | 2.27 [1.69; 3.19] | 1.87 [1.37; 2.65] |

**Supplementary Table S5**

Comparison of the discrimination (AUROC) of Model A, B and C with and without the inclusion of blood based biomarkers. In this analysis good functional outcome is defined as mRS 0-4 and poor functional outcome is defined as 5-6.

| **AUROC** | **without biomarkers** | **+ NfL** | ***p-value*** |
| --- | --- | --- | --- |
| Model A | 0.75 [0.58; 0.74] | 0.80 [0.69; 0.85] | *p=0.026* |
| Model B | 0.79 [0.66; 0.83] | 0.82 [0.73; 0.87] | *p=0.022* |
| Model C | 0.79 [0.62; 0.80] | 0.83 [0.72; 0.86] | *p=0.039* |
| **AUROC** | **without biomarkers** | **+ GFAP** | ***p-value*** |
| Model A | 0.75 [0.58; 0.74] | 0.82 [0.73; 0.88] | *p=0.002* |
| Model B | 0.79 [0.66; 0.83] | 0.83 [0.74; 0.89] | *p=0.031* |
| Model C | 0.79 [0.62; 0.80] | 0.82 [0.72; 0.88] | *p=0.067* |
| **AUROC** | **without biomarkers** | **+ NfL and GFAP** | ***p-value*** |
| Model A | 0.75 [0.58; 0.74] | 0.83 [0.75; 0.89] | *p=0.001* |
| Model B | 0.79 [0.66; 0.83] | 0.84 [0.77; 0.90] | *p=0.007* |
| Model C | 0.79 [0.62; 0.80] | 0.84 [0.76; 0.89] | *p=0.018* |

**SUPPLEMENTARY FIGURE S1**

Serum biomarkers in patients with ischemic stroke according to short-term outcome. A) Biomarker levels according to the ASPECTS on admission values and B) ASPECTS 24-72h after index stroke. NIHSS progression according to median C) serum NfL (96pg/ml) and D) serum GFAP (5.7ng/ml). E) Spearman’s correlations among biomarkers (NfL, GFAP) and NIHSS on admission. F) NIHSS change within 24 hours (h) according to median biomarker concentrations (NfL: 96pg/ml; GFAP: 5.7ng/ml). *p-value<0.05; **p-value<0.01; ***p-value<0.001; ****p-value<0.0001.

**
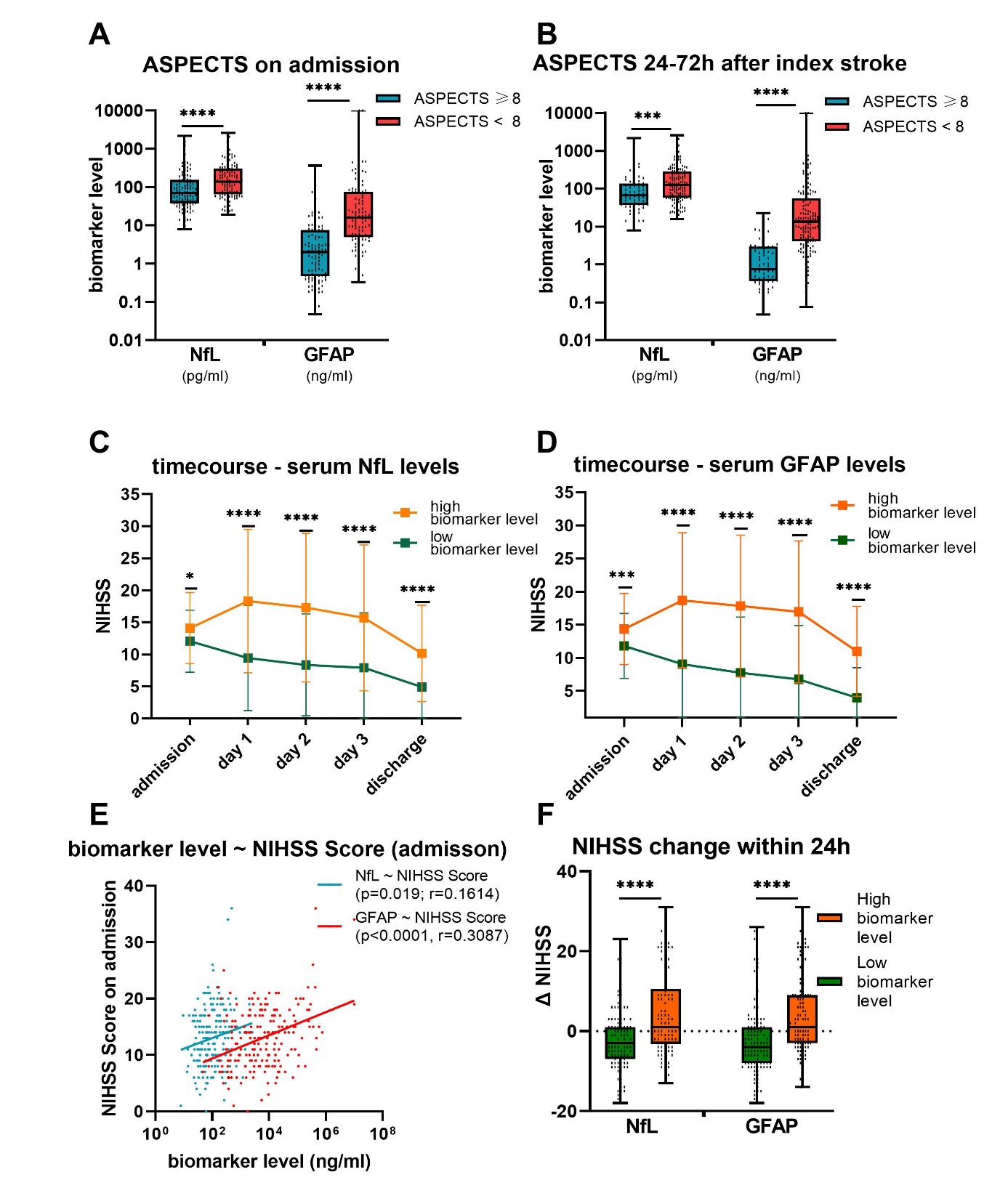
**

**SUPPLEMENTARY FIGURE S2**

Calibration plots for model C:

mRS ~ age + NIHSS on admission + ASPECTS 24-72h (8-10 vs. 0-7) + mechanical thrombectomy (yes/no)

1. (without biomarkers)
2. + **NfL**
3. + **GFAP**


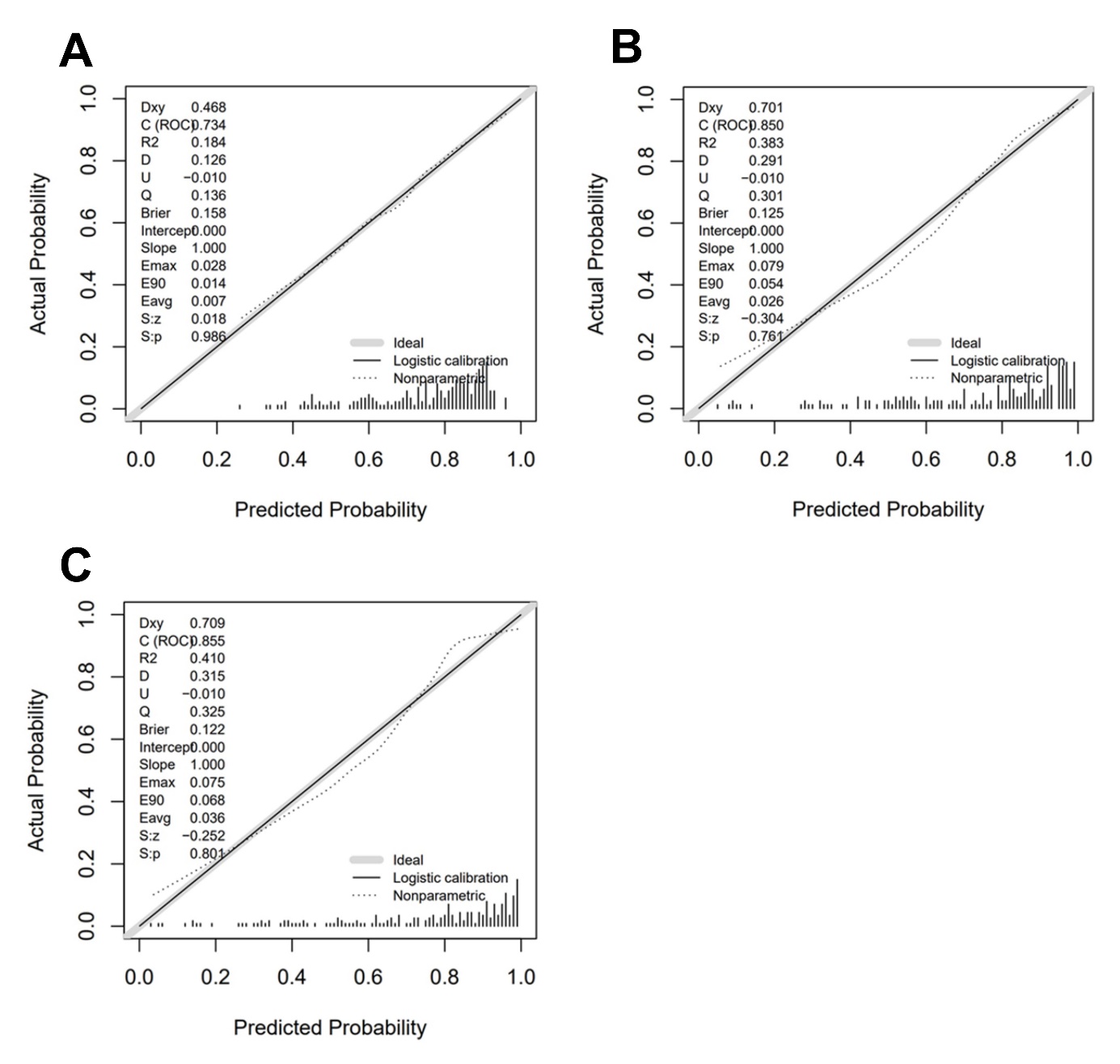


**References:**

1. Correia M, Silva I, Gabriel D, Simrén J, Carneiro A, Ribeiro S, et al. Early plasma biomarker dynamic profiles are associated with acute ischemic stroke outcomes. Eur J Neurol. 2022;29(6):1630-42.

2. Chen CH, Chu HJ, Hwang YT, Lin YH, Lee CW, Tang SC, et al. Plasma neurofilament light chain level predicts outcomes in stroke patients receiving endovascular thrombectomy. J Neuroinflammation. 2021;18(1):195.

3. Pujol-Calderón F, Zetterberg H, Portelius E, Löwhagen Hendén P, Rentzos A, Karlsson JE, et al. Prediction of Outcome After Endovascular Embolectomy in Anterior Circulation Stroke Using Biomarkers. Transl Stroke Res. 2022;13(1):65-76.
